# Multistage prediction approach of EVs charging performance in smart transportation systems by deep learning technique

**DOI:** 10.1038/s41598-025-21625-y

**Published:** 2025-10-28

**Authors:** Mustafa M. Abdelaziz, Almoataz Y. Abdelaziz, Ragab A. El-Sehiemy, Basem Abd-Elhamed Rashad

**Affiliations:** 1 Department of Electrical Power and Machines Engineering, Higher Institute of Engineering at El-Shorouk City, El-Shorouk Academy, Cairo, 11837 Egypt; 2 Department of Electrical Power and Machines Engineering, Faculty of Engineering, Ain Shams University, Cairo, Egypt; 3 Faculty of Engineering and Technology, Future University in Egypt, Cairo, 11835 Egypt; 4 Electrical Engineering Department, Faculty of Engineering, Kafrelsheikh University, KafrelSheikh, 33516 Egypt

**Keywords:** Electric vehicles (EVs), Deep learning, Charging behavior, Smart cities transportation, Electrical and electronic engineering, Energy grids and networks

## Abstract

Electric Vehicles (EVs) are increasingly recognized as a fundamental component of intelligent transportation systems within smart city frameworks. Therefore, several studies in recent decades have been trying to improve the performance of EVs to maximize the benefits from their connection to the network. Machine Learning (ML) and data-driven methods are used for analyzing EV charging behavior to maintain significant improvements in the prediction and scheduling fields. Although many of these studies have relied on historical charging data to predict the EVs’ State of Charge (SoC) and Charging Available Time (CAT), influential features have often been overlooked. These features are represented in real-time distance, road characteristics (road type, traffic pattern, and events data), and weather data. This study proposes a novel multistage approach, based on a Feedforward Deep Neural Network (FDNN) that combines historical charging data with these influential features to predict both SoC and CAT. The proposed approach outperforms existing literature with SMAPE scores of 0.00044, 0.00018 and 0.00014, 0.00012 for initial, required SoC and CAT predictions, respectively. Through comparative analyses with prior studies on the same dataset, this research highlights substantial improvements in predictive accuracy. It underscores the significance of integrating influential features for the precise prediction of EV charging behaviors within smart transportation systems.

## Introduction

Climate change and global warming have driven the rising trend of EVs. With traditional vehicles contributing significantly to high exhaust rates and carbon emissions, the widespread adoption of EVs is crucial to combat this pollution. Moreover, EVs play a vital role in peak shaving by utilizing their discharging process during peak periods. This research aims to provide a comprehensive investigation of EVs’ impact on reducing pollution levels and their effectiveness in peak shaving. The methodology involves rigorous data collection, statistical analysis, and modeling techniques to evaluate the environmental benefits and grid stability enhancement provided by EV integration; however, it is difficult to deny the continuous development of EVs in several fields. The research has begun in this field due to the importance of replacing traditional vehicles with EVs, which shows a comparison between them^1^. Then, the connection of EVs to the network is studied to determine the challenges as a result of EV integration. These challenges that appear because of the process of charging EVs are represented in increasing power losses and the peak power consumed. Also, the voltage deviation increases with the EV implementation^2^.

Although EVs are essential, challenges persist, such as EV owners depending on charging stations due to the lack of home charging opportunities for all users. Over-reliance on charging at stations causes more pressure on the network during peak times. Additionally, the integration of huge-scale EVs will impose restrictions on the networks, and Instability in power networks due to uncoordinated EV charging behavior. This research employs a methodical approach involving data analysis, and simulation studies to address these hurdles effectively. To overcome the spatial constraints limiting the expansion of charging stations for EVs, a strategic approach is imperative. Smart scheduling emerges as a viable solution, necessitating an in-depth comprehension of charging behaviors^2^. Coordinated charging entails gathering essential data such as arrival and departure times, trip frequency, distances traveled, charger type, and battery SoC. These features are meticulously analyzed in this study to devise an efficient scheduling framework. The study optimizes the utilization of existing charging infrastructure, minimizes peak power demands, and ensures EV connection to the grid without the need for extensive expansion of charging facilities.

**Fig. 1:**
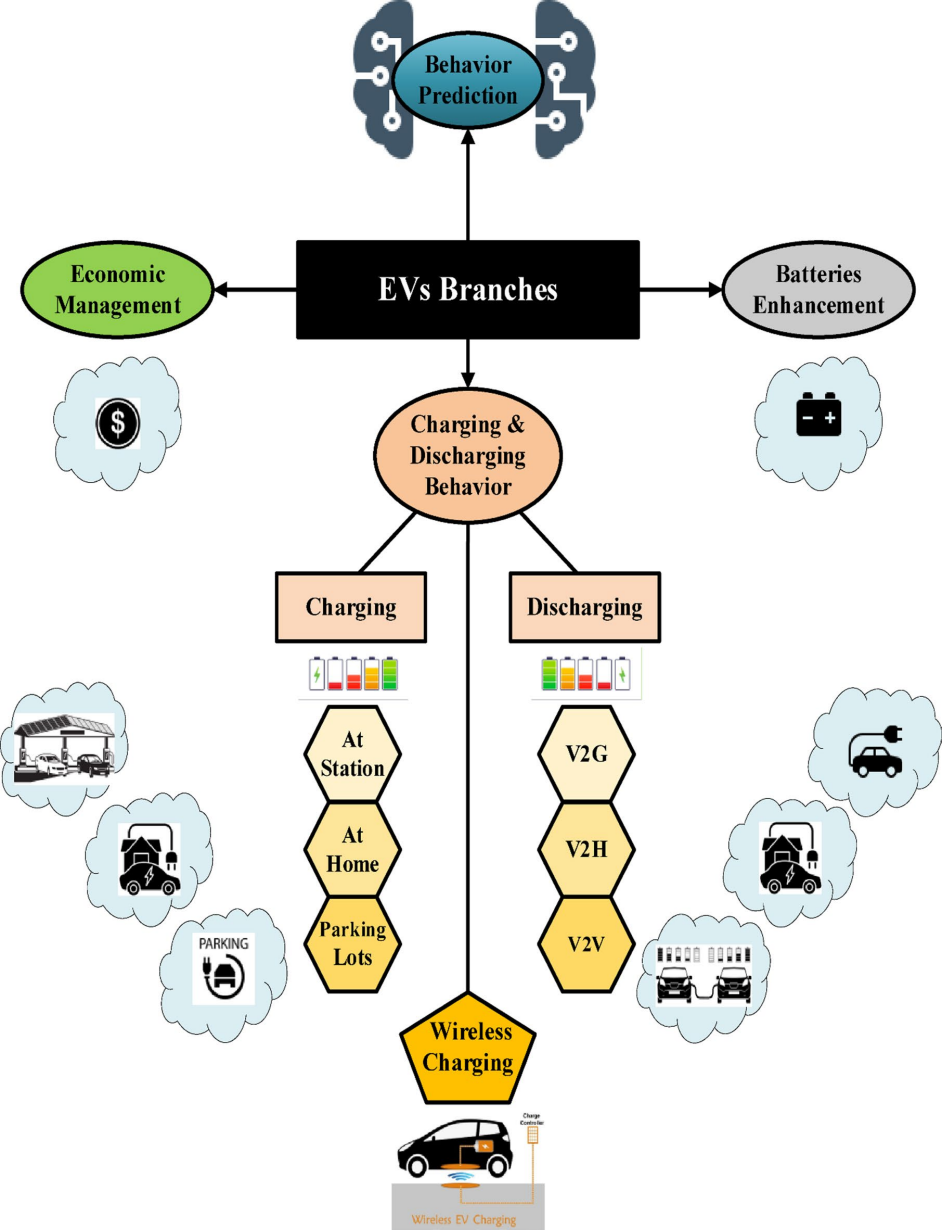
Previous EVs research classification.

The rapid advancement in EV technologies has gained significant attention from researchers and policymakers worldwide. So, frequent studies have been implemented to explore various EV aspects, including EV charging, discharging, or a combination of both^3–9^, battery development‎^10^, and economic issues‎^11^. This section aims to elucidate an overview of the existing literature on these topics, as illustrated in Fig. 1. In the rest of this section, a sample of research those are mentioned in Fig. 1 have been discussed as a general background, and then followed by the summary of related charging behavior prediction works.

### Background

Charging of EVs has emerged as a critical issue in EV topics, which directly impacts the network stability and infrastructure. Several researchers have delved into this topic, investigating different aspects of EV charging infrastructure and methods. Studies such as in^3^ demonstrate a schedule for coordinated EVs charging through actual data to maintain customer satisfaction using a genetic algorithm of shorthand a multi-objective function to a single one using weighting factors. To overcome the network limitations, the fluctuations of load have been diminished. The optimization results illustrate that the power consumed difference between peak and valley is decreased by 22% from the stochastic charging. However, the financial aspects of EV charging were not investigated in^3^.

An optimal strategy for EVs charging has been introduced in^4^, which is based on AI. It depends on fast charging to reduce the electrical network stress through the duck curve smoothing. In^5^ optimal parking lots sizing and allocation is implemented on 69-bus, 33-bus, and 9-bus networks. Also, EVs’ availability is discussed comparing with previous methods, but it requires assuming different values for EVs’ charging power to avoid the uncertainty data problem. This uncertainty arises from several assumptions, including each EV’s charging power (15 kW), annual failure rate, battery capacity of 50 kWh, and V2G dispatch time.

Also, one of the key areas of research in the field of EVs is discharging. Discharging studies focus on optimizing the use of stored energy in EV batteries, as reported in^6^. A scheduling approach for EV charging/discharging is suggested to minimize the operating cost and the peak/valley difference. EV owners have other factors besides the charging/discharging prices factor, such as arrival and departure time, which determine the availability of the charging period. So, the dynamic time-of-use price used in^6^ to ensure uncertainty is not enough for the availability of EVs to feed the load through the discharging process.

Generally, the discharging topic has been classified into vehicle-to-vehicle, vehicle-to grid, or vehicle-to home (V2V, V2G, or V2H) as in^7–9^. In^7^ a comprehensive survey of thirty studies is introduced and compared in terms of control structure and other various factors. V2G framework is proposed in^8^ to mitigate the network challenges in meeting charging demand during peak. The suggested method in^9^ achieves regulation effects such as reducing and shifting the peak period to the off-peak period. The environmental and economic issues have also been enhanced.

Battery Enhancement is another critical issue in the EV field. The performance, capacity, and lifespan of batteries significantly impact the overall efficiency and usability of EVs. Researchers are continuously working on developing new battery technologies, improving energy storage capabilities, and enhancing battery durability. These advancements aim to address the limitations of current battery technology, such as battery degradation over time, restricted driving range, and extended charging duration. A lithium-ion battery is used as a sample to evaluate the performance of the method proposed in‎^10^. This method showed promising results compared to previous research, which tested under different conditions, such as ageing, noise, and temperature impacts.

Economic considerations also play a significant role in the adoption of EVs. Researchers have examined the economic feasibility of EVs, considering factors such as purchase price, operating costs, and potential government incentives. These studies help policymakers design effective incentive programs to promote their adoption. The suggested hybrid system based on renewable energy in^11^ may reduce the cost of EV charging stations and the environmental impact.

### Related works (AI-driven EVs behavior analysis)

Recently, AI models have been used more widely in different fields to support the transition toward EV adoption. This shift aims to preserve the environment; so the challenges that affect the spread of EVs must be faced. So, research is interested in studying and predicting the EVs’ charging behavior, battery SOC, and also the spread of EVs in the market‎^12^. Some important factors affect EVs’ charging scheduling, such as weather, traffic, and predetermined and sudden events. This factor could be taken into consideration during EV behavior prediction. In^13^ charging infrastructure status for the next day could be predicted by ML. Also, the network status and high-load prices adaptation could be implemented. EVs’ travel behavior has been simulated using a multi-layer ML approach in‎^14^, where an optimal bidding model for EV service providers was also proposed.

SoC estimation has been a key focus in previous studies, such as in^15–18^. In^15^ the remaining driving range is estimated from the SOC that was predicted using SVR. MAE and R^2^ are used to evaluate the prediction results that depend on the dataset of EV drivers for two weeks. SOC prediction based on EVs battery historical dataset is proposed in‎^16^, where Mileage and EVs battery voltage, current, and temperature are used to train the LightGBM model. These EV batteries’ data were also used as input data for the prediction models that were implemented in‎^17,18^. The model of the LSTM neural network model with MLP that was executed in^17^ was evaluated through one evaluation parameter of MSE. The same parameter MSE is used beside MAE, and RMSE to evaluate the three implemented models of SVM, KNN, and GPR in‎^18^. In^[‎19^ extreme ML is proposed for online prediction of the lithium batteries’ SOC.

A four-time series is produced in^20^ by a Python-based tool that produces BEV profiles. It is called emobpy, which is based on empirical mobility statistics and customizable assumptions. EV mobility is the first time series produced that depends on some factors, such as driver type, daily trip number, and departure and step time. Trip location, destination, duration, and distance per trip must also be available. This first time series is used with driving electricity consumption, the second time series, charging station availability, and the charging strategy as the emobpy input to get the grid electricity demand for the fourth time series.

A model based on physics and graph attention has been suggested in^21^ to enhance the prediction of EVs charging demand under the dynamic price situations, which was evaluated through the use of over 18 thousand EVs as a dataset. It is undeniable that studying and predicting the impact of EV charging on the grid to avoid potential grid problems. Therefore, the power consumption of EV charging stations has been predicted through three different models in^22^, for two different states. Also, the charging station operation cost could be obtained from these prediction results.

Table 1 summarizes the important parameters for the related previous works on EV Behavior prediction. Moreover, a comprehensive comparison table is provided, which contrasts our proposed approach with a set of recently published studies on EV charging behavior prediction. This table highlights key aspects such as the methodologies employed, datasets used, performance metrics, and external factors. The comparison clearly demonstrates the strengths of our multistage deep learning approach, particularly in its ability to integrate both historical and real-time data, including distance, road characteristics, and weather data. In contrast, our approach achieves superior predictive accuracy, as evidenced by the remarkably low SMAPE scores for SoC and CAT predictions. This comparison underscores the novelty and effectiveness of our method, positioning it as a significant advancement in the field of EV charging performance prediction.

### Research gap and paper contribution

The proposed work covered some of the weak points of previous EV research. These points can be summarized as:-.

- EVs charging scheduling depending on unrealistic data as outlined in‎^5^.
- Not-applicable assumption for long parking period as reported in^23^.
- Neglecting the departure time in the charging scheduling process^23^.

The results for EV parameters prediction have been enhanced using the proposed prediction models. Although previous studies have applied ML for predicting state of charge, session duration and energy consumption, they primarily focused on using Historical Charging Data (HCD). But sometimes additional features were also incorporated. Motivated by these approaches, this work investigates the use of additional input features to spot their impact on the prediction accuracy.

The key contributions of this work are as follows:

1. A novel approach is proposed for predicting EV charging behavior (SoC and CAT) that incorporates HCD, traffic, and weather data.
2. A novel implementation-based FDNN architecture is proposed for SoC and CAT estimation.
3. Performance evaluation indices, i.e. Symmetric Mean Absolute Percentage Error (SMAPE), Mean Absolute Error (MAE), Mean Squared Error (MSE), Root Mean Square Error (RMSE), and coefficient of determination (R^2^) are presented for comparison.
4. The empirical analysis demonstrates that the proposed work, which incorporates additional data, significantly enhances the prediction accuracy compared with previous studies, which relied solely on historical charging information.

### Paper organization

The rest of the paper is organized as follows: Sect. 2 provides a detailed clarification of the proposed methodology, starting with a general overview of the proposed approach, followed by a description of the FDNN structure, and concluding with a discussion of the four-stage implementation. This is followed by the results, which are outlined and evaluated in Sect. 3. Section 4 presents the results’ discussion and comparison, while the conclusion is illustrated in Sect. 5.

**Table 1:** Comprehensive comparison between the proposed prediction approach and previous related works^13–20^.

Paper	Year	Dataset	Features	Model	Prediction	Appraisal	Evaluating Metrics
Ref. ‎^12^	2023	357 EVs monthly sales dataset	EVs safety specifications Other EVs specifications	LSTM ConvLSTM Hybrid LSTM with two-dimensional Attention and Residual network	EVs sale estimation EVs share in each segment Factors affecting EVs sales recognition	Motivate the EVs Market Lack of data needed to improve prediction results due to the unavailability and inaccessibility of this data, like the warehouses EVs number.	MAPE, NRSME_range, NRSME_mean, R^2^, slope, & intercept of fitted linear regressions
Ref. ‎^13^	2021	Charging station occupation dataset (2019) Tragic dataset (2021)	Weather Traffic	Average Week Gradient Boosting Classifier Random Forest Classifier	Charging station availability	Lake of traffic availability of data.	Accuracy, AUC, Recall, Precision, F1, Kappa, & MCC
Ref. ‎^14^	2020	Weekends and weekdays historical data	Current chain start time & start Signal	Multi-layer ML Algorithm	Travel chain type Current chain end time Next chain start time Travel distance	The EVs data is required to be updated as they were old (2008–2009).	Not mentioned
Ref^15^.	2023	EVs drivers real data	EVs battery Data (V, I, T). Driver speed. Ambient temperature.	Support Vector Regression (SVR) with a Radial Basis Function (RBF)	SoC RDR	PDR is estimated not predicted. Data was collected for short period of 2 weeks.	MAE R^2^
Ref^[‎16^.	2024	EVs battery historical data	EVs battery Data (V, I, T). Mileage.	LightGBM Extra Tree Regressor (ETR)	SoC	Ignoring features that increase the accuracy of the results, such as weather conditions.	RMSE MAE MSE R2
Ref^17^.	2025	Real-life battery Discharge simulation datasets	EVs battery Data (V, I, T). charge/discharge cycles No.	LSTM Neural Network Model with MLP	SoC	Need to increase the evaluating metrics.	MSE
Ref. ‎^18^	2024	Custom EVs dataset	EVs battery Data (V, I, T). Humidity. Ambient temperature. Motor temperature.	SVM ANN GPR	SoC		RMSE MAE MSE
Ref^19^.	2023	UCI datasets	Not mentioned	SNN LSTM GRN	SoC	Need to increase the evaluating metrics.	RMSE
Ref^[‎20^.	2021	German mobility data	EVs data Charging station data Charging strategy	Emobpy	EVs mobility Driving electricity consumption Grid availability Grid demand	The research contributes significantly to providing the datasets that is used in the prediction as implemented in our proposed work.	
Proposed Prediction Approach	2025	Weekdays EVs real data	Distance Road characteristics Weather	FDNN	SoC CAT	Integration of heterogeneous data sources. Study the impact of each feature separately. Contribution of advancement of smart transportation systems through a robust framework for EV SoC and CAT prediction.	SMAPE RMSE MSE MAE R^2^

PDR: Remaining Driving Range, GBM: Gradient-Boosting Methodology, V: Voltage, I: Current, T: Temperature, LSTM: Long Short-Term Memory, MLP: Multilayer Perceptron, SVM: Support Vector Machine. ANN: Artificial Neural Network, GPR: Gaussian Process Regression, AUC: Area under the receiver operating characteristic curve, Kappa: Cohen’s kappa score, MCC: Matthews correlation coefficient, UCI: University of California - Irvine, SNN: Sparse Neural Network, GRN: Gated Recurrent Neural.

## The methodology of the proposed prediction approach

**Fig. 2:**
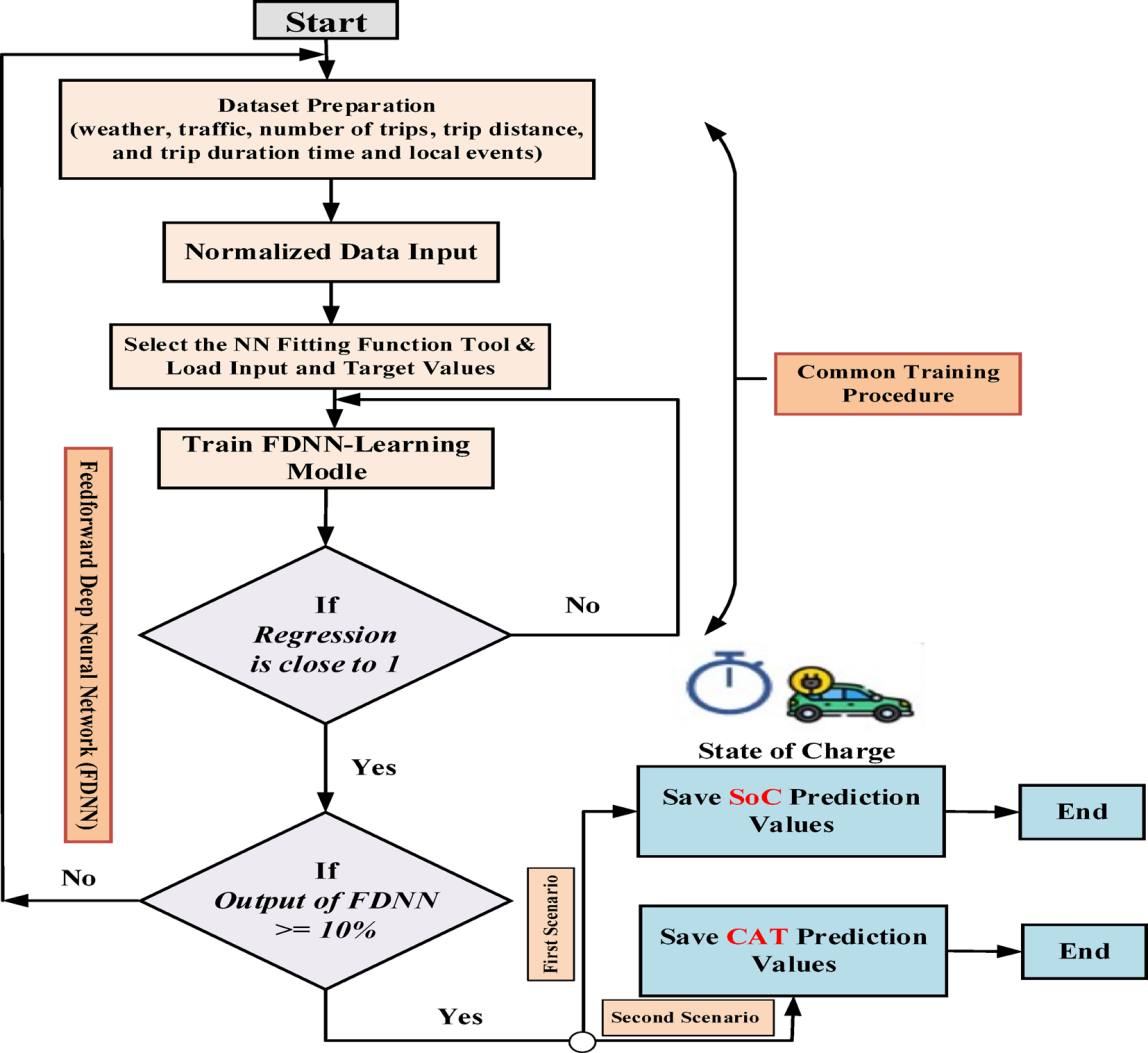
Overall flowchart implementation of the proposed FDNN-based prediction approach.

The overall flowchart in Fig. 2 clarifies the implementation of the proposed FDNN prediction approach. The suggested prediction approach strategy utilizes a single FDNN. The goal of this network is to develop a prediction approach by accepting as input the normalized data of the selected BEV, including distance, road characteristics, and weather data.

The selected BEV model is the Tesla Model 3 (TM3). The nominal battery capacity ($\:{N}_{Battery}=78.1\:kWh$) is derived from manufacturer data^24^; all battery data are illustrated in Table 2. The EV charging scenarios have three main probabilities, which are charging at home, at stations, or in parking lots. The scenario of charging at home is the basis for the proposed work, where the charging period is the period between the arrival and departure times. TM3 can be charged according to manufacturing data by using a regular socket or a charging station. Charging time depends on the maximum EV’s capacity and the charging station features. EV charging differs by country; some countries use 1-phase connections to the network, while others use a 3-phase connection.

Table 3 illustrates the indications of the actual driving range under different conditions. The worst scenario represents the cold weather based at 10 °C, which requires heating. Hot weather of 35–40 °C, such as that in Egypt, is also considered among the worst scenarios. The mild weather is the greatest scenario based on 23 °C, which does not need A/C. A fixed speed of 110 km/h is assumed for the highway. The real range will depend on speed, driving mode, weather, and road type^24^.

**Table 2:** Tesla model 3 battery data^24^.

Battery Item	Data
Nominal Capacity (kWh)	78.1
Operating Capacity (kWh)	75
Battery	Lithium-ion
Cathode Material	NCM
Cells Number	4416
Pack Configuration	96s46p
Architecture (V)	400
Nominal Voltage (V)	357

**Table 3:** Tesla model 3 battery data^24^.

Road type	Weather	Distance (km)
City	Mild	700
Highway	Mild	460
Combined	Mild	560
City	Cold Hot	455
Highway	Cold Hot	350
Combined	Cold Hot	400

### The predictive scenarios studied

One of the most important parameters for EV charging scheduling is SoC. The proposed model is created to predict both the initial SoC (SoC-In) and required (SoC-Req). In addition to other parameters, arrival and departure times have been predicted, which are known as CAT. The following two scenarios illustrate the target for each prediction model, which is affected by the number of trips and the total distance for each EV.

#### First scenario (3 inputs & 2 outputs)

The first model is implemented using FDNN, which depends on three main input parameters to predict the values of SoC-In and SoC-Req. The three input parameters are the total distance for each EV trip number, weather temperature, and road type. The weather is classified as moderate or not, where the immoderate temperature represents the hot or cold weather. The road type may be city, highway, or combined road. Road type affects the speed, which also directly affects the energy consumption. So, the selected EV model data explain the differences in the total mileage according to the weather and road type, as previously mentioned in Table 3.

**Fig. 3:**
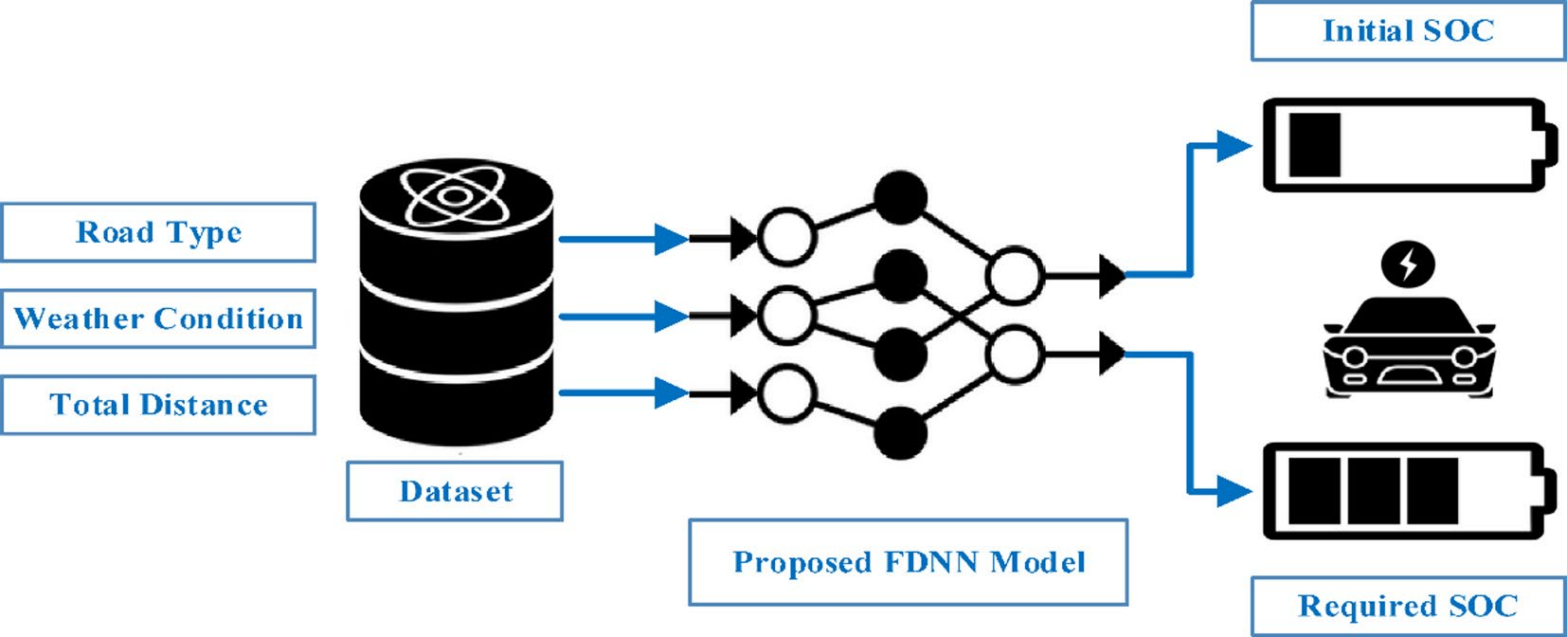
First scenario of 3 inputs & 2 outputs.

The initial SoC is predicted according to the total distance of trips on the previous day. However, the required SoC depends on the total distance of trips on the next day for each EV. The number of trips and distance of each trip have been stochastically distributed for all EVs according to the percentage in‎^20^. The road type and the weather condition represented in the ambient temperature are used as inputs with the total distance to enhance the model prediction results. Figure 3 illustrates the sequence of the proposed prediction model for the first scenario.

#### Second scenario (3 inputs & 4 outputs)

**Fig. 4:**
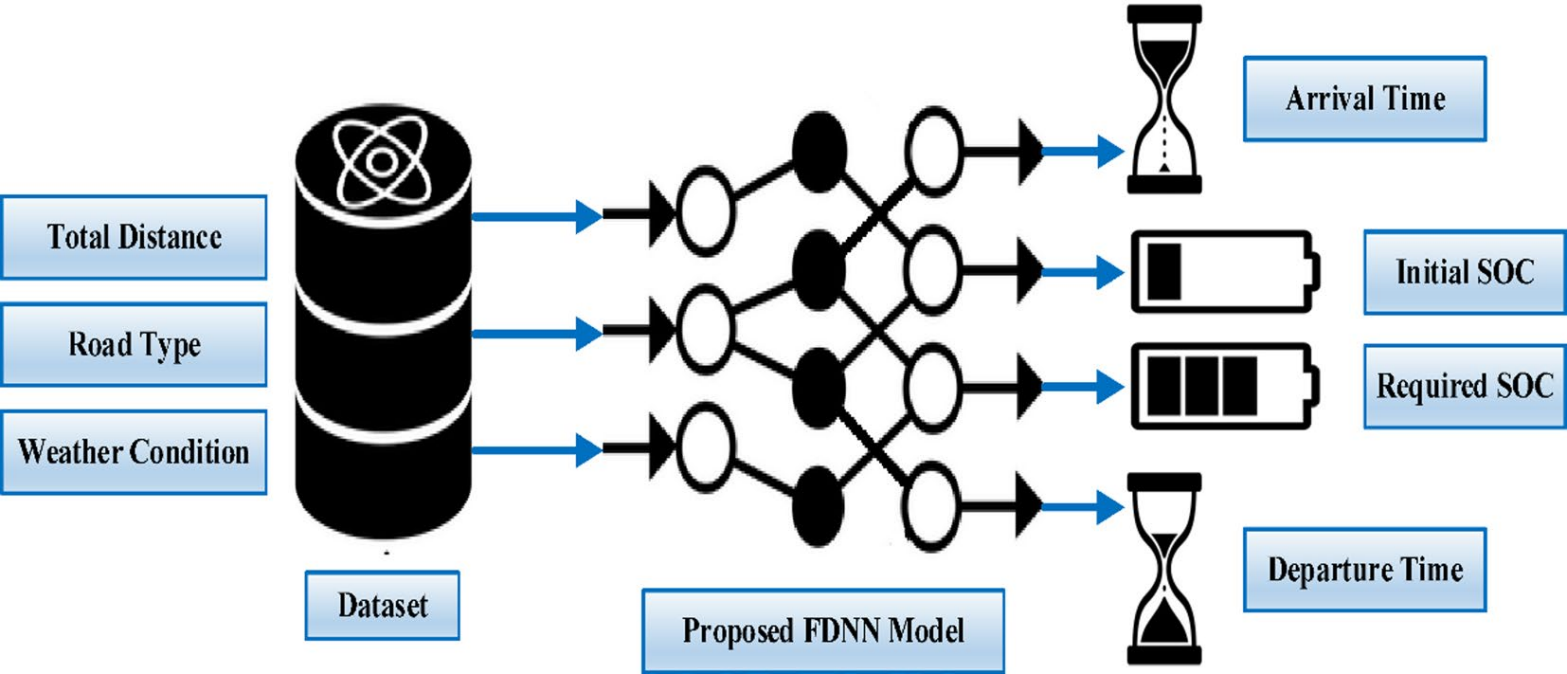
Second scenario for 3 inputs & 4 outputs.

In this scenario, the predicted target is represented in the initial and required SoC, in addition to arrival and departure time. The same inputs of the first scenario are also used here to train the second model. The sequence of the second proposed model is illustrated in Fig. 4.

### Detailed description of FDNN structure

An artificial neural network with more than one hidden layer of neurons between the input and output is referred to as a DNN^25,26^. ANN is a computational model capable of performing both ML and pattern recognition tasks^27^. DNNs are used to simulate complex nonlinear systems. Moreover, DNN computation is efficient because it involves solving basic algebraic equations. This feature enables DNNs to address issues promptly^28–30^.

**Fig. 5:**
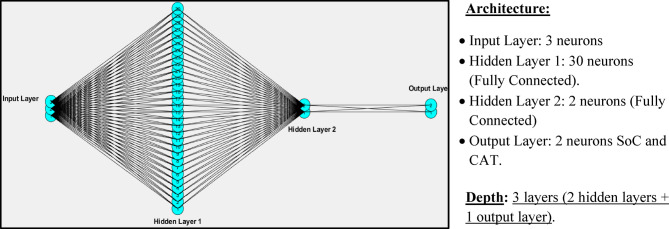
Visual representation of the suggested FDNN.

The FDNN used in this study comprises an input layer with three neurons representing the features: distance, road characteristic, and weather data. Two fully connected hidden layers were implemented, consisting of 30 and 2 neurons, respectively, and activated using ReLU functions. The output layer includes two neurons corresponding to the predicted outputs: SoC and CAT, as illustrated in Fig. 5.

This multi-layered structure ensures non-linear feature extraction and robust learning capabilities, distinguishing it from simpler ML models. Unlike conventional ML models such as linear regression or single-layer perceptron’s, the FDNN architecture leverages multiple hidden layers and non-linear activation functions to capture complex relationships between diverse input features and output predictions. This enables superior generalization across heterogeneous input data. An FDNN can be considered a DNN under certain conditions. Specifically, the classification depends on the depth of the network. When the FDNN contains more than two hidden layers, it is classified as a DNN. The term “deep” reflects the increased depth of the network, which enables it to model complex data patterns and hierarchies. The distinction lies primarily in the depth of the architecture, not in the forward-pass structure of the network itself, as both shallow and deep networks can be feedforward in nature.

The premise behind the proposed FDNN prediction approach is that input features can quickly reveal their impact on the prediction accuracy. These input features include distance (Total distance for each EV’s trips), road characteristics, and weather data. Road characteristics are represented by road type, which could be city, highway, or combined roads, in addition to traffic patterns and events data. The weather data are represented by the ambient temperature, which could be mild or not mild (hot or cold). These data are fed into an FDNN for predicting charging behavior. The FDNN-based prediction approach is trained through these input data to predict EV parameters, which are the initial SoC (SoC-In), the required SoC (SoC-Req), and the charging available time.

Finally, the information produced can be utilized in decision-making processes for subsequent control operations, such as predicting the initial and required SoC and CAT. A FDNN consists of four main types of layers: input, hidden, softmax, and output layers. These layers are commonly used in data-driven prediction and diagnostic approaches. To confirm that all values lie within the range [0, 1], feature scaling is applied as follows, where (*P*) is the input vector: -

1 $$\:P{\prime\:}=\frac{P-min\left(P\right)}{{max\:}\left(P\right)-{min}\:\left(P\right)}$$

The following nonlinear transformation is used in the hidden layers to transform the input data into high-dimensional features. Here, $\:x=(2,\dots\:,d)$, $\:\bar{Y}\:$is the hidden vector, $\:\bar{y}\:$ is the bias vector, $\:W$ is the weight matrix, and $f$ is the activation function applied element-wise. The output of the final hidden layer is transformed using Eq. (2) without using the activation function given in Eq. (3).

2 $$\: \begin{array}{*{20}c} {\bar{Y}}_{1}=\:f\:({W}_{1\:}.P+\:\bar{y}\:) \\ {\bar{Y}}_{x}=\:f:({W}_{x\:}.{\bar{Y}}_{x-1}+\:{\bar{y}}_{x}\:) \end{array}$$

3 $$\:{\bar{Y}}_{s}=\:{W}_{s\:}.{\bar{Y}}_{d}+\:{\bar{y}}_{s}$$

The softmax function is used to determine the output value of each neuron, as in Eq. (4). Then the label with the highest output value is selected as the predicted label for the input data.

4 $$\:{Q}_{j}=\frac{{e}^{{\bar{Y}}_{s,j}}}{\sum\:_{j=1}^{{n\bar{Y}}_{s}}{e}^{{\bar{Y}}_{s,j}}}$$

In the proposed work, the FDNN is used as a prediction framework. Its role is demonstrated through a comprehensive four-stage implementation, where each stage builds on the previous one to produce more reliable predictions, as outlined in the following sections.

### FDNN prediction approach implementation

**Fig. 6:**
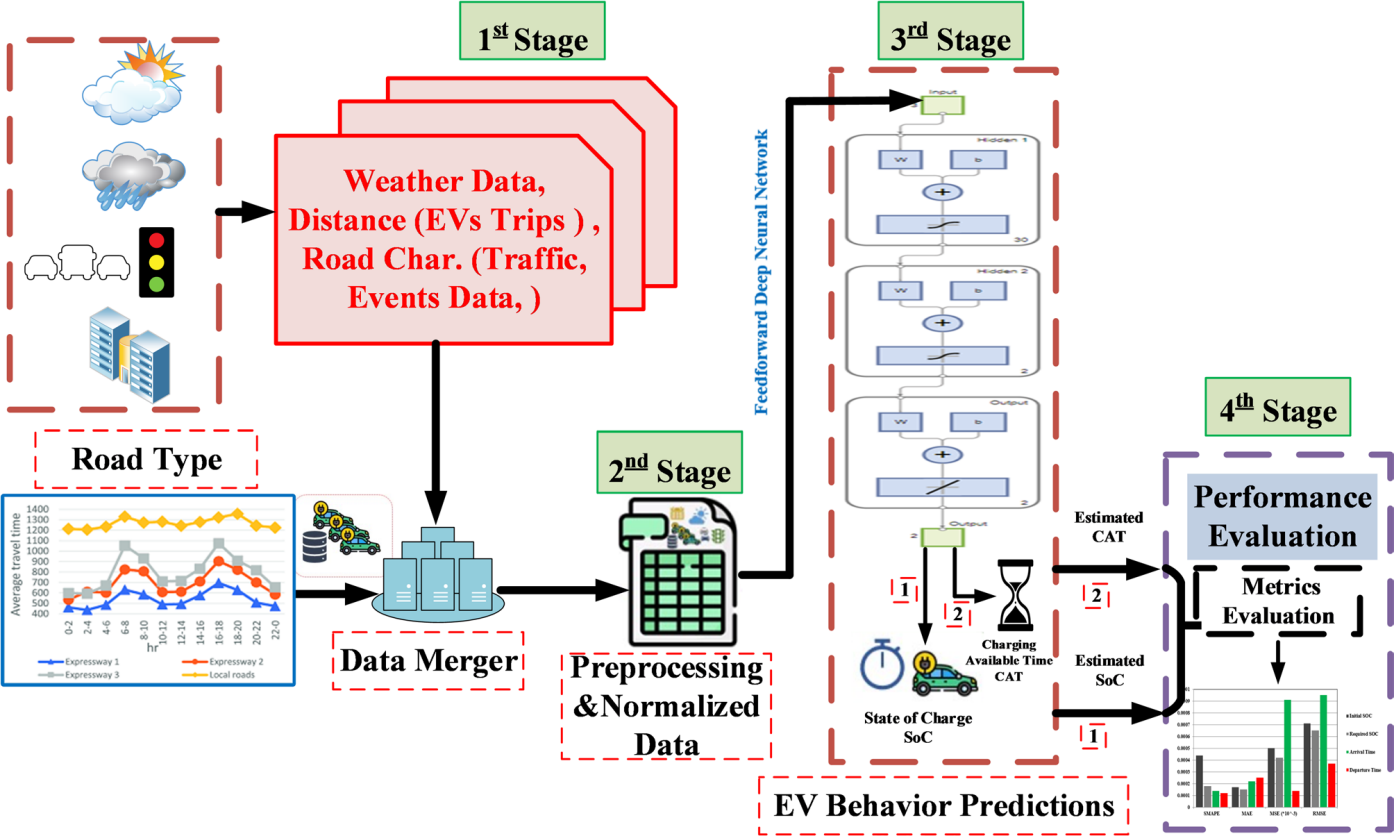
Structure of FDNN-based prediction approach.

The proposed FDNN general structure is illustrated in Fig. 6, which consists of four stages:

- 1 st Stage: Dataset preparation.
- 2nd Stage: Input data preprocessing and normalization.
- 3rd Stage: Training of the feedforward deep neural network.
- 4th Stage: Performance metrics evaluation.

#### 1 st stage: dataset preparation

The data taken from‎^20^ is used to generate a dataset for 1000 EVs. Also, we can produce data for any EVs sample size. These data are represented in the number of trips, trip distance, and trip duration time for each EV. Table 4 illustrates the percentage value of each EV’s trip number for both working and weekend days. These percentages are then applied to the 1000 EV dataset for the working-day scenario only, as it is considered more critical than the weekend case, as shown in Table 5.

**Table 4:** EVs for trips no. probability per working and weekend days.

No. of Trips	Working Day EVs No. (%)	Weekend Day EVs No. (%)
0	35.4	50.7
1	0	0
2	29.9	27.5
3	8.3	4.4
4	12.5	10.2
5	13.9	7.2

**Table 5:** EVs trips no. per working and weekend days for 1000EVs.

No. of trips	Working Day EVs No. (EV)
0	354
1	0
2	299
3	83
4	125
5	139

After that, the number of trips is distributed for the whole number of EVs (*N* = 1000 EVs) randomly. The number of trips for working and weekend days are 2042, and 1450 respectively. The total number of trips is estimated using Eq. (5) for working days. The duration of each trip is distributed according to the trip distance of each EV. Then the distance and duration time are also randomly distributed according to Eq. (6).

5 $$\:{{TT}_{N}}_{1}=\sum\:_{t=0}^{5}{(t}_{n}*{{EV}_{n}}_{1}\%*N)/100$$

6 $$\:{{T}_{N}}_{1}=\left({DDEV}_{n}\%*N*{{TT}_{N}}_{1}\right)/\left(100*N\right)$$

*where*:

##### $\:{{TT}_{N}}_{1}$

Total No. of working days trips.

##### $\:{t}_{n}$

Trips No.

##### $\:{{EV}_{n}}_{1}\%$

Working days EVs No. for each number of trips as percentages.

##### $\:N$

Total EVs No.

##### $\:{{T}_{N}}_{1}$

Trips No. for working days at specific distances and times.

#### 2nd stage: input data preprocessing and normalization

To generate a superior training environment, utilizing multiple datasets is highly effective. Six distinct cases for data input are employed using a combination of datasets and training models. The preprocessing of data involves cleaning and preparing the collected data by neglecting faulty data, outliers and inconsistencies, to enhance the model performance. In this work, we used standardization to convert the data to date-time objects to obtain the weather and road type for a particular charging record. This approach allows for the easy extraction of relevant information. Rather than determining the traffic level at a definite time, it considered the total traffic time through the day, enabling the model to identify the influence of traffic levels on charging performance. As well as performing normalization to confirm that the data are on a consistent format and scale, as given by Eq. (2).

**Fig. 7:**
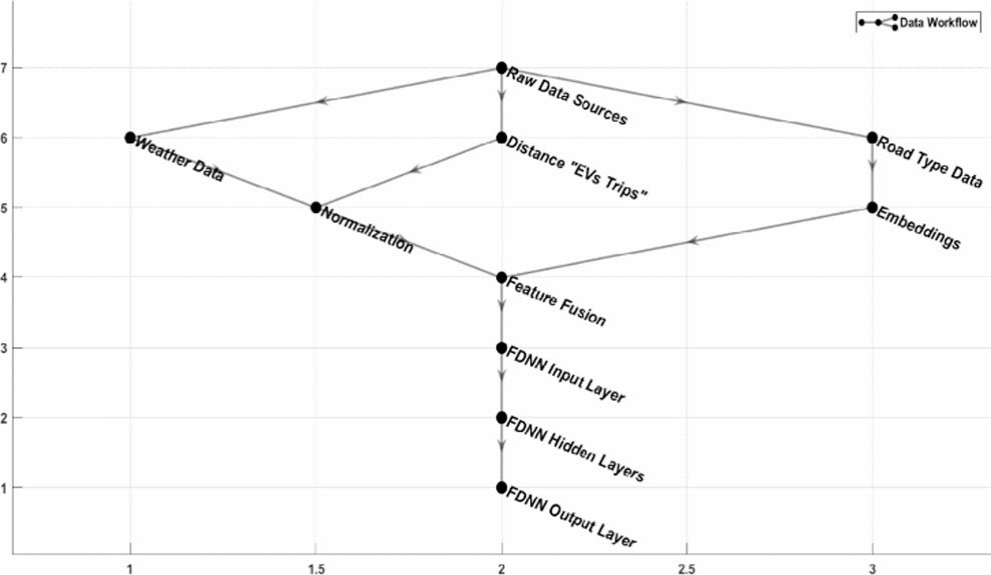
Data processing workflow for FDNN.

The systematic approach used to integrate diverse data types ensures that the FDNN learns meaningful feature interactions rather than relying on simple concatenation. The process begins with a robust preprocessing pipeline, where continuous variables such as distance, road characteristics, and weather data are normalized to a uniform scale, and categorical variables like road types are encoded using methods such as embeddings. Embeddings, in particular, transform categorical data into dense numerical representations, enabling the model to capture complex relationships between categories, such as urban versus rural road characteristics. This fusion of data streams occurs at the input layer of the FDNN, where the architecture is designed to facilitate interaction among the preprocessed features. A detailed workflow diagram showcasing the preprocessing steps and a schematic representation of the FDNN’s structure are illustrated in Fig. 7, highlighting how the network processes and combines features to predict SoC and CAT.

#### 3rd stage: training of FDNN for proposed prediction approach

**Fig. 8:**
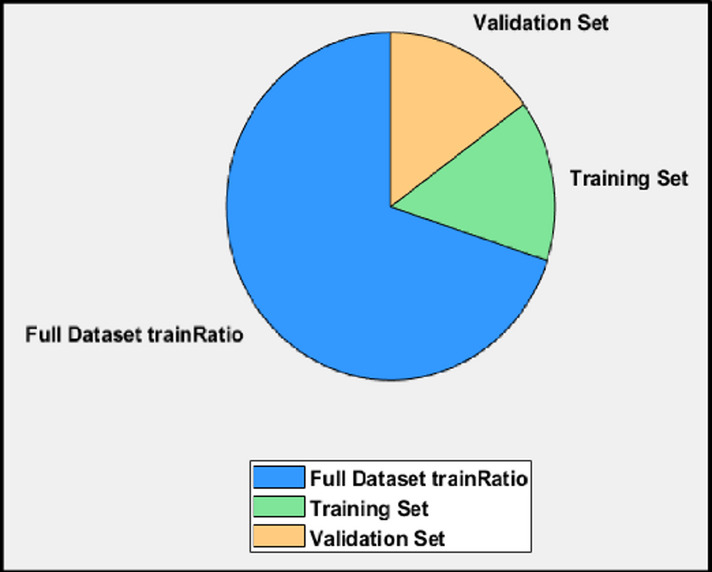
Data partitioning workflow.

The design of the FDNN structure requires careful identification of the type and number of layers, the number of neurons in each layer and the activation function used. In the proposed approach, a total of 1000 samples were considered for the FDNN design.70% (700 samples) were used for training, 15% (150 samples) were used for testing, and 15% (150 samples) were used for validation. Figure 8 illustrates how the data were split into training, validation, and test sets. Common architectures for prediction approach tasks include Convolutional Neural Networks (CNNs) and Recurrent Neural Networks (RNNs), such as LSTM networks.

**Fig. 9:**
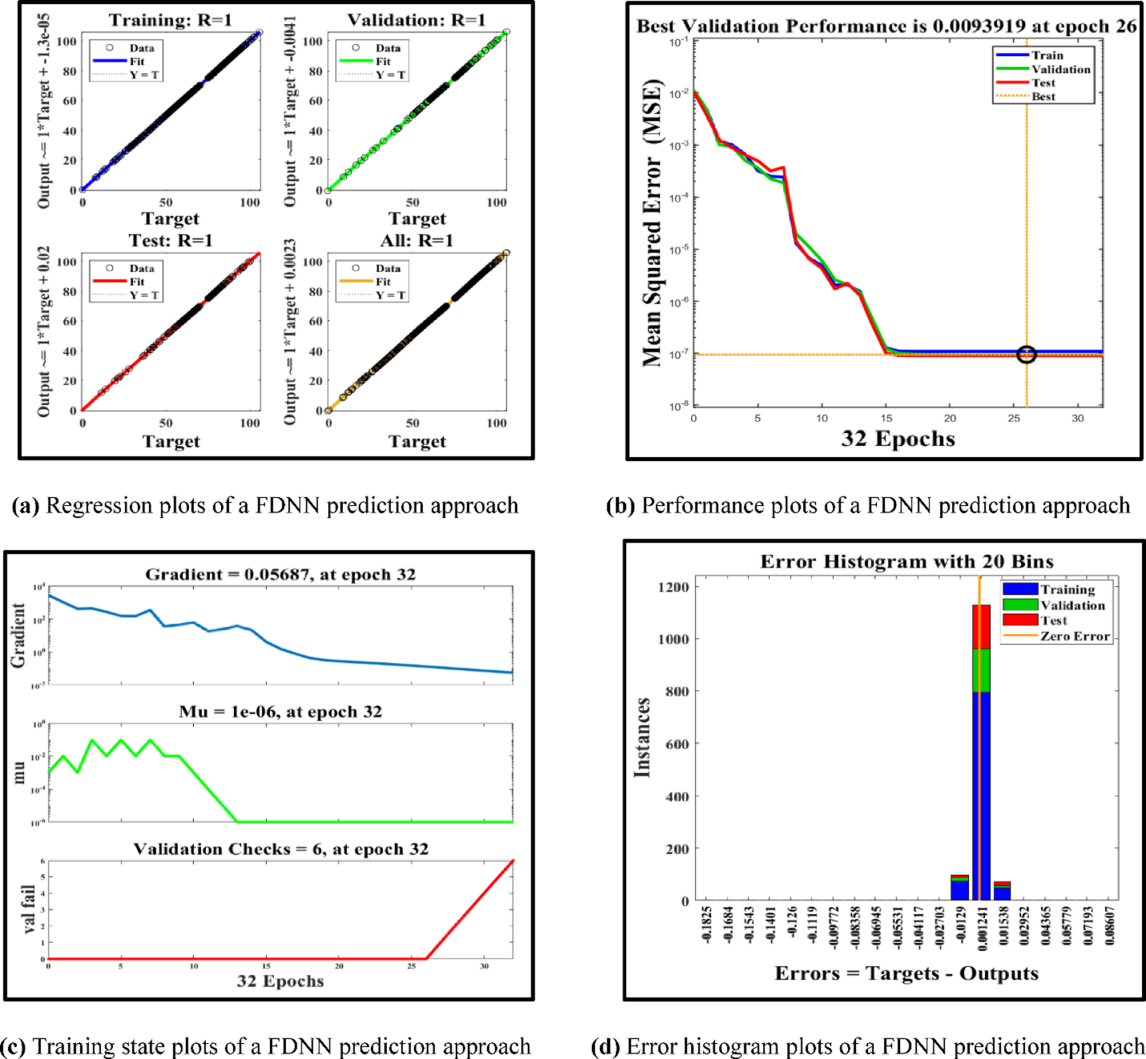
Regression, performance, training state, and error histogram plots of FDNN-based prediction approach.

Figure 9 shows the performance of the FDNN prediction model. From the regression plot shown in Fig. 9a, it is noticed that the regression value is equal to 1, indicating that the FDNN is accurately trained to identify the prediction values under study. The MSE is also very low, further demonstrating the model’s precision.

**Fig. 10:**
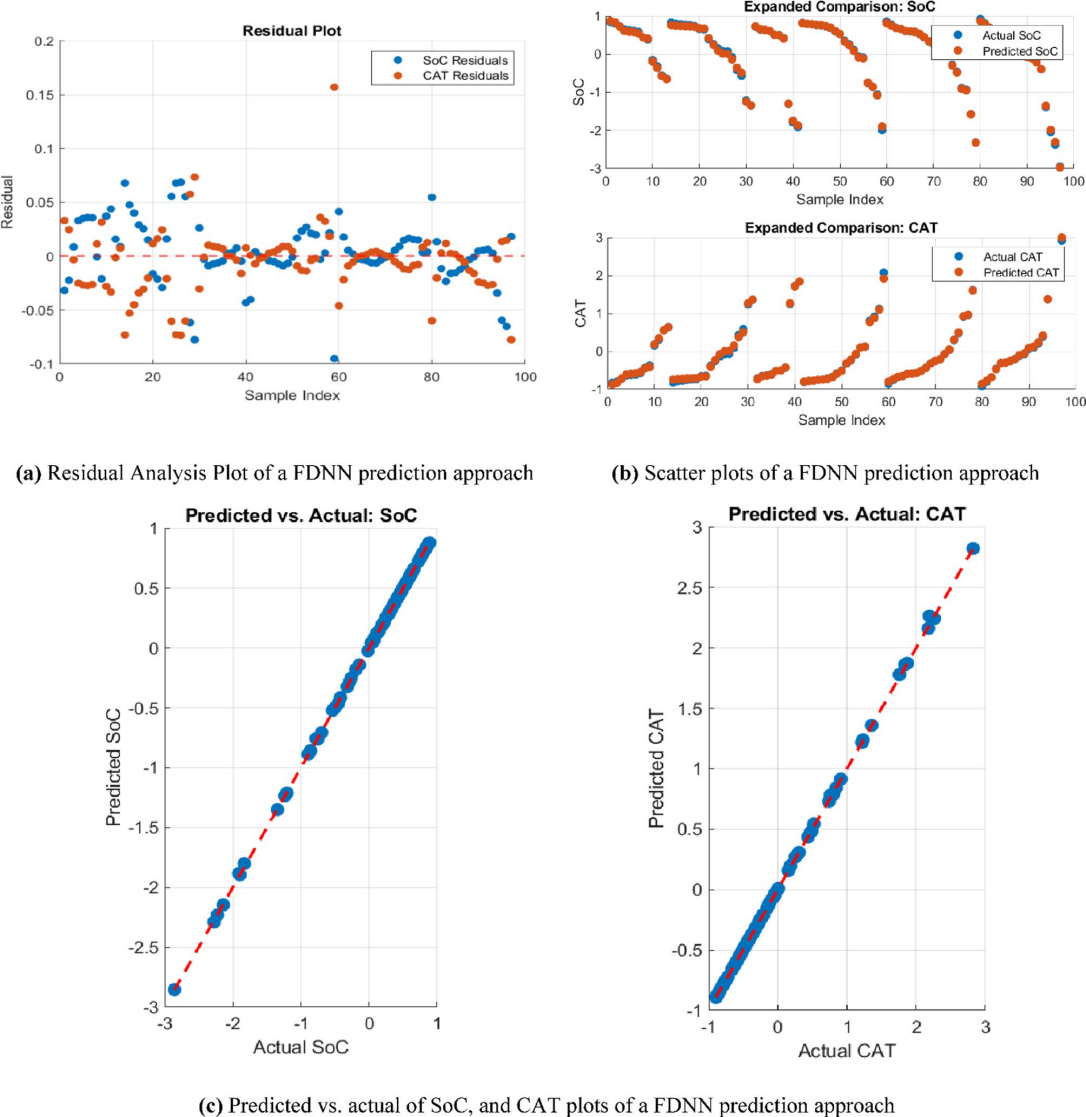
Residual analysis, scatter plots, and predicted vs. actual of SoC, and CAT plots of FDNN-based prediction approach.

Moreover, Fig. 10 provides visual validation of the model’s performance, addressing the error metrics and model accuracy. Figure 10a provides the residual clustering plot, which illustrates the differences between predicted and actual values, with residuals tightly clustering around zero. This indicates minimal bias and suggests that the model accurately captures the relationships in the data without significant under-fitting or over-fitting. Figure 10b provides the scatter plot comparing predicted and actual values for both SoC and CAT. Points clustering closely along the ideal diagonal line signify high predictive accuracy. Separate plots for SoC and CAT further highlight the model’s ability to handle multiple output variables effectively. Figure 10c provides the predicted vs. actual value plots, showcasing the robustness of the model across diverse scenarios.

For SoC, the plot demonstrates consistency in accurately predicting battery states, which is crucial for EV efficiency. For CAT, the plot emphasizes reliable time estimations, which are critical for planning charging schedules. Together, these plots validate the model’s ability to generalize across varying input conditions. They provide strong evidence against over-fitting and substantiate the low error values reported, ensuring the reliability and practical applicability of the predictions in real-world scenarios. To address potential over-fitting, we employed cross-validation (5-fold cross-validation) and included regularization techniques such as dropout layers in the FDNN architecture to confirm that the low error metrics are consistent across different subsets of the data. The results confirm that the exceptionally low error values are due to the model’s high precision and not over-fitting.

#### 4th stage: performance metrics evaluation

To evaluate the performance of predictions made by the FDNN model, various metrics are utilized, as discussed in^10^. In this study, five measures are defined, which are commonly used in related works to assess the SoC and CAT prediction results for the proposed FDNN model. Equations (9)–(13) outline the metrics used to evaluate this work, which are applied accordingly.

(i) Symmetric mean absolute percentage error (SMAPE):

9 $$\:SMAPE=\:\frac{1}{k}\:\sum\:_{i=1}^{k}\frac{\left|{Y}_{a\left(i\right)}-{Y}_{p\left(i\right)}\right|}{(\left|{Y}_{a\left(i\right)}\right|+\:\left|{Y}_{p\left(i\right)}\right|)/2}*100\%$$

(ii) Mean absolute error (MAE):

10 $$\:MAE=\frac{1}{k}\sum\:_{i=1}^{k}\left|{Y}_{a\left(i\right)}-{Y}_{p\left(i\right)}\right|$$

(iii) Mean Squared Error (MSE):

11 $$\:MSE=\frac{1}{k}\sum\:_{i=1}^{k}{\left({Y}_{a\left(i\right)}-{Y}_{p\left(i\right)}/\:{Y}_{a\left(i\right)}\right)}^{2}$$

(iv) Root mean square error (RMSE):

12 $$\:RMSE=\sqrt{\sum\:_{i=1}^{k}{({Y}_{a\left(i\right)}-{Y}_{p\left(i\right)})}^{2}\:/\:k}$$

(v) Coefficient of determination (R^2^):

13 $$\:{R}^{2}=1-\frac{\sum\:_{i=1}^{k}{({Y}_{a\left(i\right)}-{Y}_{p\left(i\right)})}^{2}}{\sum\:_{i=1}^{k}{({Y}_{a\left(i\right)}-\tilde{a})}^{2}}$$

In this context, $\:{Y}_{a}$ is the real value while the predicted value is$\:\:{Y}_{p}$, $\tilde{a}$ is the mean of real values, and $\:k$ denotes the groups of values in the dataset. Lower scores for RMSE, MAE, and SMAPE indicate accurate predictions, which occur when the predicted value $\:{Y}_{p}$ is very close to the actual value$\:\:{Y}_{a}$​. The R^2^ value measures the goodness of fit for regression and typically ranges between 0 and 1. A score of 1 indicates perfect predictions, with higher values representing better performance^30^. The subsequent two sections present and analyze the results of the proposed approach, followed by a comparison with prior studies to demonstrate the approach’s effectiveness and contributions.

## Results and evaluation metrics

The results have been evaluated using various metrics such as SMAPE, MAE, MSE, RMSE, and$\:\:{\text{R}}^{2}$. Equation (9) to (13) outline the metrics used in this work, and these performance metrics will be evaluated accordingly. Table 6 illustrates the evaluation for the first and second proposed scenarios. The results of the first model are distinguished from the second model by a slight difference in accuracy due to several factors. In the first model, two parameters are deduced from three inputs, while in the second model, four parameters are deduced from the same three inputs. Therefore, to improve the results of the second model, more inputs can be added, or the outputs can be obtained separately by predicting the initial and required SoC as in the first scenario. Then, predict the other parameters of arrival and departure time.

**Fig. 11:**
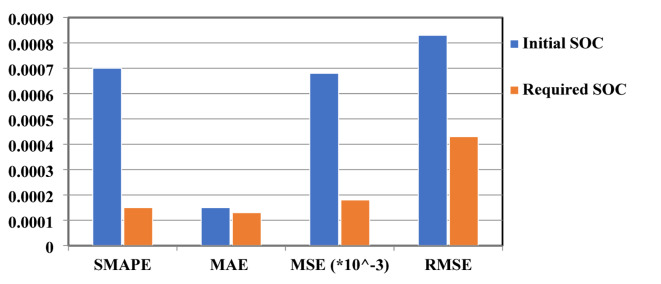
The evaluation metrics values of the first scenario results.

**Fig. 12:**
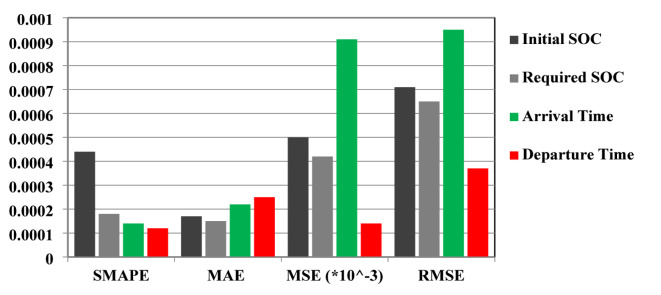
The evaluation metrics values of the second scenario results.

Tables 7 and 8 show a sample of results, representing the predicted and targeted values. The initial and required SoC are the target outputs for the first scenario, while the second scenario has the target outputs of initial and required SoC, in addition to the charging available time. The weather and road conditions are illustrated in Tables 7 and 8 for each SoC value. The evaluation of the first scenario results is summarized in Fig. 11, while the evaluation of the second scenario results is summarized in Fig. 12.

**Table 6:** The first and second scenarios evaluation results.

Scenarios	First scenario		Second scenario			
Index	Initial SoC	Required SoC	Initial SoC	Required SoC	Arrival time	Departure time
SMAPE	0.0007	0.00015	0.00044	0.00018	0.00014	0.00012
MAE	0.00015	0.00013	0.00017	0.00015	0.00022	0.00025
MSE	6.8*10^−7^	1.8*10^−7^	5*10^−7^	4.2*10^−7^	9.1*10^−7^	1.4*10^−7^
RMSE	0.00083	0.00043	0.00071	0.00065	0.00095	0.00037
R^2^	1	1	1	1	1	1

**Table 7:** A sample of predicted and targeted values for the first scenario.

Distance	Road Type	Weather	Initial SoC			Required SoC		
			Actual	Predicted	Error	Actual	Predicted	Error
58	Combined	Not Mild	61.714	61.714	0.0000398	78.625	78.625	−0.00027
140	City	Mild	58.333	58.333	0.00012	80.000	80.000	0.000119
162	Combined	Not Mild	46.857	46.857	0.000233	85.125	85.125	−0.00039
222	City	Mild	51.500	51.500	−0.00016	82.929	82.929	0.000053
248	Highway	Mild	39.000	39.000	−0.00030	88.053	88.053	−0.00032
266	City	Mild	47.833	47.833	−0.00015	84.500	84.500	−0.00014
291	Highway	Not Mild	21.500	21.499	0.001477	95.786	95.786	−0.00034
304	City	Mild	44.667	44.666	0.000231	85.857	85.857	−0.00014
312	Highway	Not Mild	18.000	17.997	0.003237	97.286	97.287	−0.00106
331	Combined	Not Mild	22.714	22.714	0.00013	95.688	95.688	−0.00027

**Table 8:** A sample of predicted and targeted values for the second scenario.

Distance	Road Type	Weather	Initial SoC			Required SoC		
			Actual	Predicted	Error	Actual	Predicted	Error
35	City	Mild	67.083	67.083	−0.00005	76.25	76.25	0.00067
64	Highway	Mild	62.000	62.000	0.000146	78.000	78.000	−0.00079
126	Combined	Mild	57.400	57.400	0.000105	80.478	80.478	−0.00021
210	City	Not Mild	43.750	43.750	−0.00028	86.667	86.667	0.000101
259	Highway	Not Mild	26.833	26.833	−0.00016	93.500	93.500	0.000242
323	Combined	Not Mild	23.857	23.857	0.00069	95.188	95.188	0.00027

Distance	Road Type	Weather	Arrival Time			Departure Time		
			Actual	Predicted	Error	Actual	Predicted	Error
35	City	Mild	10.000	9.9998	0.0002	200.00	200.00	0.00013
64	Highway	Mild	13.800	13.798	0.002	190.00	190.00	−0.0007
126	Combined	Mild	19.800	19.800	−0.0001	201.60	201.60	0.0001
210	City	Not Mild	27.000	27.000	0.00017	200.00	200.00	−0.00023
259	Highway	Not Mild	32.000	32.000	−0.00022	201.20	201.20	0.00008
323	Combined	Not Mild	38.000	38.000	0.0004	197.00	197.00	−0.00036

## Discussion and comparison

**Fig. 13:**
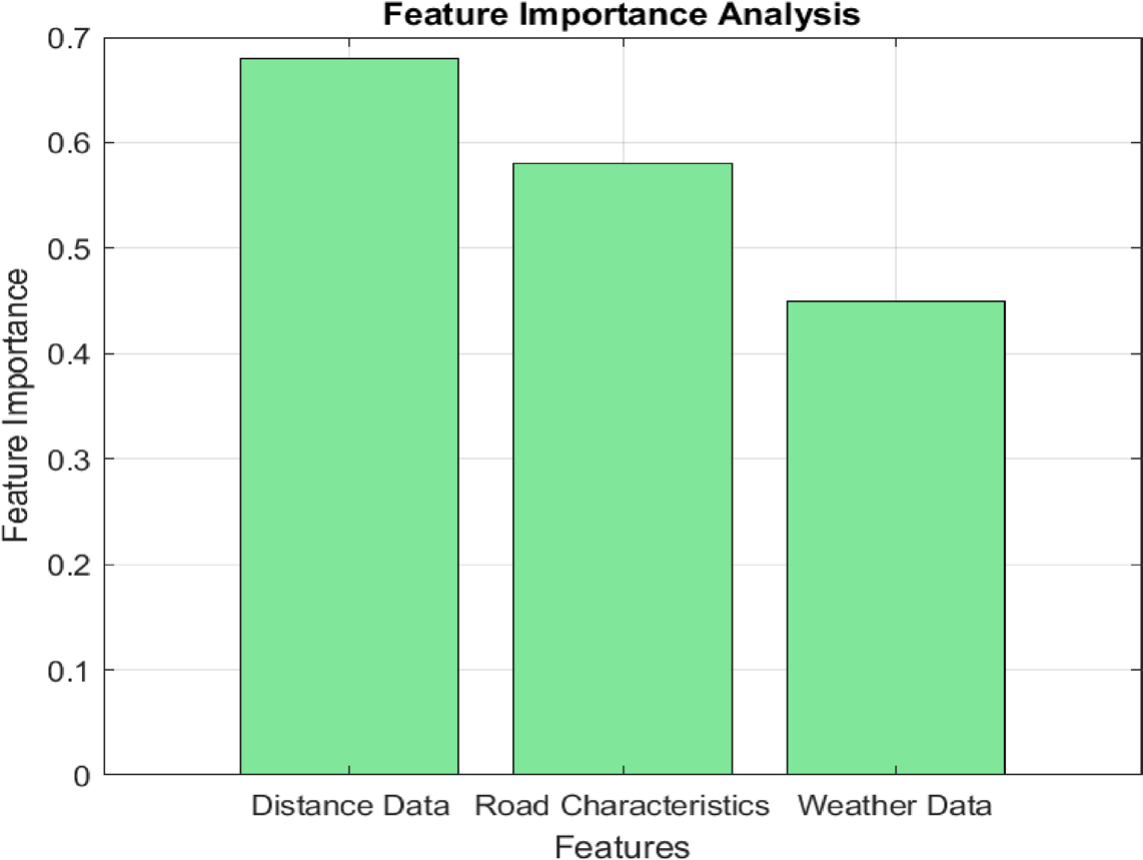
Feature importance for predictive accuracy.

A systematic ablation study to evaluate the contribution of each input feature to the predictive accuracy of the model has been implemented. This process involved sequentially removing one input feature at a time and observing the resulting performance metrics, such as SMAPE, MAE, and RMSE, to determine how each feature impacts the overall prediction of the SoC and CAT. Table 9 summarizes the required SoC results of the second scenario, showing how the removal of individual features affects the model’s performance. Figure 13 illustrates a bar chart that shows normalized importance scores for each feature in terms of their contribution to SMAPE reduction.

**Table 9:** The impact of features on the required SoC prediction in the second scenario.

Feature Set	SMAPE	MAE	RMSE	*R*²
All Features	0.0001800	0.0001500	0.0006500	0.9999
Without Weather Data	0.0003150	0.0002367	0.0008912	0.9277
Without Road Characteristics	0.0003749	0.0002133	0.0009331	0.9175
Without Distance Data	0.0004550	0.0002733	0.0011000	0.8971

The illustrated results in Table 9 and Fig. 13 show the effect of each feature, highlighting the importance of incorporating all contextual features to achieve robust prediction performance. The impact of these features can be formulated as follows:

- *Distance and road characteristics* These factors had the most significant impact on predictive accuracy, particularly for the required SoC.
- *Weather and traffic patterns* These factors were less influential individually but contributed to overall model performance when combined with other features, particularly for CAT.
- *Events data* While events data had a smaller impact, it improved prediction accuracy in specific scenarios.

Additionally, scatter plots comparing predicted and actual values were used to visually validate the model’s performance as previously illustrated in Fig. 10, which validates model accuracy by showing how well predictions align with actual values. These Figures clearly indicate a consistent predictive ability across the test dataset. Also, to address potential over-fitting, we employed cross-validation and included regularization techniques such as dropout layers in the FDNN architecture. The results confirm that the exceptionally low error values are due to the model’s high precision, not over-fitting.

When comparing the proposed prediction approach across various metrics, considering the overall R^2^ and the SMAPE, it appears that predicting the SoC and CAT is particularly challenging. Moreover, across different scenarios, we observed that users’ self-predictions of their behavior often differed significantly from their actual performance, underscoring the need for predictive analytics. Compared to previous works, the results of this study outperformed all prior studies reporting similar evaluation metrics^31–35^.

Table 10 summarizes the results from these prior works in comparison to those achieved in this study. Specifically, for session duration, our results are more accurate than those in^31^. However, it is important to note that all previous work used a different dataset from the one used in this study, making direct comparisons potentially unsuitable. Nonetheless, when keeping the comparison within the same dataset, it is evident that the inclusion of additional road type and weather led to an enhancement in EVs’ charging performance prediction.

**Table 10:** Comparison between the proposed prediction approach and previous works.

Source	Prediction	Model	Features	Results				Year
First Proposed Prediction Approach	Initial SoC Required SoC	FDNN	Distance, Road Characteristics, and Weather Data	Index	**SoC-In**		**SoC-Req**	2025
				SMAPE	0.0007		0.00015	
				MAE	0.00015		0.00013	
				MSE	6.8*10^−7^		1.8*10^−7^	
				RMSE	0.00083		0.00043	
				R^2^	1		1	
Second Proposed Prediction Approach	Initial SOC Required SOC Arrival Time Departure Time	FDNN	Distance, Road Characteristics, and Weather Data	Index	**SoC-In**		**SoC-Req**	2025
				SMAPE	0.00044		0.00018	
				MAE	0.00017		0.00015	
				MSE	5*10^−7^		4.2*10^−7^	
				RMSE	0.00071		0.00065	
				R^2^	1		1	
Ref.^31^	SoC	ANN SVM Linear GPR Ensemble Boosting Ensemble Bagging	Battery Capacity Battery Voltage Battery Current and Battery Temperature	MSE	0.00054			2021
				MAE	0.00027			
				RMSE	0.02329			
				R^2^	0.999			
Ref.^32^	SL, Energy Consumption	RF, SVM, XGBoost, & ANN	HCD, Weather, Traffic, Events Data	SMAPE	9.92%			2021
				MAE	66.5 min			
				SMAPE	11.6% Consumption			
				R^2^	0.7			
Ref.^33^	Departure Time	XGBoost	HCD, Vehicle Type, Charging Location	MAE	82 min			2020
Ref.^34^	Energy Requirements	XGBoost	HCD	MAE		4.6 kWh		2020
				R^2^		0.52		
Ref.^35^	SL, Energy Consumption	Ensemble Model of SVM, RF, & DKDE	HCD	SMAPE		10.4% Duration		2019
						7.5% Consumption		

Finally, the FDNN’s novelty lies in its systematic data integration approach and specialized architecture, aspects that cannot be fully represented by comparing it to generic models. Highlighting its unique design and performance metrics adequately fulfills the study’s objectives. The research emphasizes the integration of diverse data types (e.g., distance, road characteristics, and weather data) and demonstrates its impact on prediction accuracy, rather than benchmarking against general models that may not be specialized for this context. Compared models may not be explicitly designed for the specific task of predicting both SoC and CAT, and adapting them could lead to suboptimal configurations or unfair evaluations. The effort to align existing models with the task might dilute the uniqueness of the proposed FDNN approach.

## Conclusion

In the proposed work a framework is presented for predicting the most important EV charging behaviors related to scheduling, specifically EV state of charge and charging available time (arrival and departure times). Unlike previous studies, we incorporated additional features, such as distance, road, and weather data, in addition to HCD. FDNN was trained to predict charging behavior and examined how the hidden layers and the number of neurons in the final hidden layer impact the network’s performance. Furthermore, the limitations of our approach can be mitigated by employing careful feature selection and leveraging domain expertise to address these constraints effectively.

The first model was trained using three inputs to predict the initial and required SoC only. The results of this model were evaluated using accuracy and error metrics, showing promising outcomes. Specifically, the MSE was$\:\:{1.8\text{*}10}^{-7}$, the MAE was 0.00013, and R² is unity. The second model also demonstrates promising results, consistent with the evaluation factors mentioned in previous works. The prediction performance of our models is superior to that reported in earlier studies. Furthermore, we have achieved significant improvements in predicting charging behavior using the HCD, which demonstrates the potential of incorporating distance, road characteristics, and weather data information into charging behavior prediction.

The future extension of this work can be considered as developing new models of ML methodologies with more features, such as an in-depth study of the factors affecting EVs’ performance. Consequently, the EV’s performance impacts on vehicle consumption and changes in initial and final SoC values. These factors include road angle, wind direction, and resistance. Furthermore, these methodologies could be applied to various types of EVs and compared to help users choose the most suitable type.

## Data Availability

The data supporting this study’s findings are available from the corresponding author upon reasonable request.

## Abbreviations

EVs: Electric vehicles
ML: Machine Learning
SoC: State of Charge
BEV: Battery Electric vehicles
DNN: Deep Neural Network
CAT: Charging Available Time
V2V: Vehicle-to-Vehicle
FDNN: Feedforward Deep Neural Network
MAE: Mean Absolute Error
V2H: Vehicle-to-Home
RMSE: Root Mean Square Error
V2G: Vehicle-to-Grid
SMAPE: Symmetric Mean Absolute Percentage Error
MSE: Mean Squared Error
KNN: K-Nearest Neighbours
R^2^: Coefficient of Determination
CNNs: Convolutional Neural Networks
RF: Random Forest
LSTM: Long Short-Term Memory
AI: Artificial Intelligence
SoC-In: Initial State of Charge
SVM: Support Vector Machine
HCD: Historical Charging Data
ANN: Artificial Neural Network
SL: Session length
RNNs: Recurrent Neural Networks
TM3: Tesla Model 3
SoC-Req: Required State of Charge
